# *Sox7* is dispensable for primitive endoderm differentiation from mouse ES cells

**DOI:** 10.1186/s12861-015-0079-4

**Published:** 2015-10-16

**Authors:** Masaki Kinoshita, Daisuke Shimosato, Mariko Yamane, Hitoshi Niwa

**Affiliations:** Laboratory for Pluripotent cell studies, RIKEN, Centre for Developmental Biology, 2-2-3, Minatojima-Minamimachi, Chuo-ku, Kobe, Hyogo 650-0047 Japan; Laboratory for Development and Regenerative Medicine, Kobe University Graduate School of Medicine, 7-5-1 Kusunokicho, Chuo-ku, Kobe, Hyogo 650-0017 Japan; Present address: Department of Pluripotent Stem Cell Biology, Institure of Molecular Embryology and Genetics, Kumamoto University, 2-2-1 Honjo, Chuo-ku, Kumamoto 860-0811 Japan

**Keywords:** ES cells, XEN cells, primitive endoderm, Sox7

## Abstract

**Background:**

Primitive endoderm is a cell lineage segregated from the epiblast in the blastocyst and gives rise to parietal and visceral endoderm. *Sox7* is a member of the SoxF gene family that is specifically expressed in primitive endoderm in the late blastocyst, although its function in this cell lineage remains unclear.

**Results:**

Here we characterize the function of *Sox7* in primitive endoderm differentiation using mouse embryonic stem (ES) cells as a model system. We show that ectopic expression of *Sox7* in ES cells has a marginal effect on triggering differentiation into primitive endoderm-like cells. We also show that targeted disruption of *Sox7* in ES cells does not affect differentiation into primitive endoderm cells in embryoid body formation as well as by forced expression of *Gata6*.

**Conclusions:**

These data indicate that *Sox7* function is supplementary and not essential for this differentiation from ES cells.

**Electronic supplementary material:**

The online version of this article (doi:10.1186/s12861-015-0079-4) contains supplementary material, which is available to authorized users.

## Background

Mouse blastocysts at E4.5 consist of three cell types: epiblast, primitive endoderm and trophectoderm. The epiblast is composed of pluripotent cells that give rise to all embryonic lineages in later developmental stages [1]. In contrast, both primitive endoderm and trophectoderm form extra-embryonic parts such as the yolk sac and placenta, respectively. Primitive endoderm differentiates into two types of endoderm after implantation. One is the parietal endoderm (PE) that migrates along the mural trophectoderm and covers its inner surface to form the Reichert membrane. The other is the visceral endoderm (VE) that covers the outer surface of epiblast and extraembryonic ectoderm derived from trophectoderm. PE cells show mesenchymal cell-like characteristics such as stellate morphology, weak cell adhesion and rapid migration ability. In contrast, VE cells show typical epithelial morphology with tight cell adhesion.

Transcription factors (TFs) have pivotal roles in determining cell fates in developmental processes. In pre-implantation embryos, the Gata family zinc-finger transcription factor, Gata6, appears to be the primary TF that determines primitive endoderm fate as it is expressed at the earliest time point (E2.5) [2] among the primitive endoderm-specific TFs, and *Gata6*-null embryos fail to form functional visceral endoderm [3]. In addition, recent reports showed that *Gata6* deficient blastocyst-stage embryos fail to form primitive endoderm before implantation [4, 5]. The family member Gata4 is co-expressed in the primitive endoderm [6] and possibly shares function with Gata6. *Gata4*-null embryos die around E9.5 with both primitive and definitive endoderm and heart defects [7, 8]. However, chimeric complementation of the extraembryonic lineage with wild type cells, allows contribution of *Gata4*-null ES cells to cardiac and definitive endoderm cell lineages without abnormality [9], suggesting that its importance in proper extraembryonic endoderm development is limited. In addition, the involvement of the group F Sox family members, Sox7 and Sox17, is suggested by their expression in the primitive endoderm [10, 11] as well as the inability to derive extraembryonic endoderm (XEN) stem cells from *Sox17*-null blastocysts [11]. *Sox17*-null embryos show a defect in the primitive endoderm lineage only in the diapause situation [10, 12]. These findings suggest that their roles are not in the formation, but rather the maturation to PE and VE. *Sox7*-null embryos have recently been reported to have a lethality phenotype before E14.5 with heart development failure [13], further suggesting a redundant role in primitive endoderm development. Detailed analyses of the expression patterns of these four transcription factors in wild-type and mutant embryos supports a model of sequential Gata6 → Sox17 → Gata4 → Sox7 transcription factor activation within the primitive endoderm lineage [10], although the precise function of *Sox7* in this process is unclear.

In addition to XEN cells, embryonic stem (ES) cells derived from pre-implantation stage epiblast provide a powerful tool to analyze the functions of transcription factors in determining cell fates. We have previously reported that forced expression of either *Gata4* or *Gata6* in ES cells triggers their differentiation to primitive endoderm cells that exhibit the characteristics of XEN cells in their morphology, gene expression patterns and their ability to contribute to PE after blastocyst injection [14, 15]. *Shimoda et al.* reported that over-expression of *Sox17* in ES cells was not able to induce differentiation but rather facilitated the differentiation of the primitive endoderm that spontaneously differentiated toward PE and VE cells on the surface of an ES cell aggregate, embryoid body (EB). [16]. They also reported that *Sox17*-null ES cells showed a defect in maturation of PE and VE in EBs, suggesting a role for *Sox17* in late stages of extraembryonic endoderm development. A similar defect was observed in EBs made with *Gata4*-null ES cells [17, 18]. Therefore, in vitro differentiation systems of ES cells are regarded as good models of primitive endoderm differentiation, and allow the assessment of the gene function involving in the process [19].

Here we report the function of *Sox7* in the context of differentiation of primitive endoderm cells derived from ES cells. We find that inducible expression of *Sox7* causes marginal differentiation of ES cells towards primitive endoderm, and that *Sox7*-null ES cells normally generate primitive endoderm cells in EBs and differentiate to XEN cells by the activation of Gata6. These results indicate that Sox7 function is not essential for either differentiation to primitive endoderm or for maturation to PE or VE.

## Results

### Parallel up-regulation of *Sox7*, *Sox17* and *Gata4* is triggered by the artificial activation of Gata6 in ES cells

**Fig. 1:**
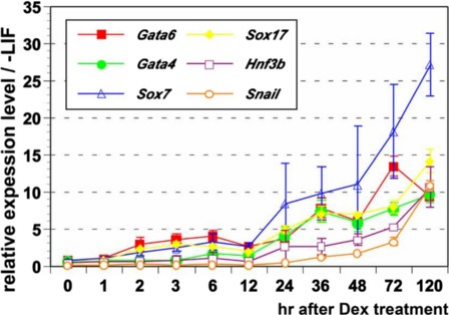
Up-regulation of extraembryonic endoderm-associated transcription factor genes after induction of Gata6GR. The expression levels of extraembryonic endoderm-associated transcription factor genes were estimated by qPCR analysis in 5G6GR ES cells carrying *Gata6GR* after Dex treatment and the relative expression levels normalized by *Gapdh* were shown along the time course. The level of expression of each transcript in EB3 ES cells cultured without LIF for 120 hours was set at 1.0. Error bars indicate standard deviation (n = 3)

We previously reported that artificial induction of Gata6 transcriptional activity using a chimeric transgene composed of full-length mouse *Gata6* and human *glucocorticoid receptor ligand-binding domain* (*G6GR*), induces homogeneous differentiation of mouse ES cells into XEN-like cells when their nuclear localization is induced with dexamethasone (Dex) [15]. To investigate the sequential activation of other TFs expressed in the primitive endoderm during mouse development, we first performed qPCR analysis along the time-course of differentiation after addition of Dex to the medium. *Sox7* and *Sox17,* as well as the endogenous *Gata6,* started to be up-regulated within 2 hours after addition of Dex while *Gata4* remained at the basal level (Fig. 1). At 24 hours after the addition of Dex, all 4 of these TFs were dramatically up-regulated as well as other TFs such as *Hnf3b/Foxa2* and *Snail* (Fig. 1). These data suggested that both *Sox7* and *Sox17* could be direct targets of Gata6 in mediating its function of triggering differentiation toward primitive endoderm.

### Forced expression of *Sox7* in ES cells shows marginal impact on differentiation to XEN-like cells

**Fig. 2:**
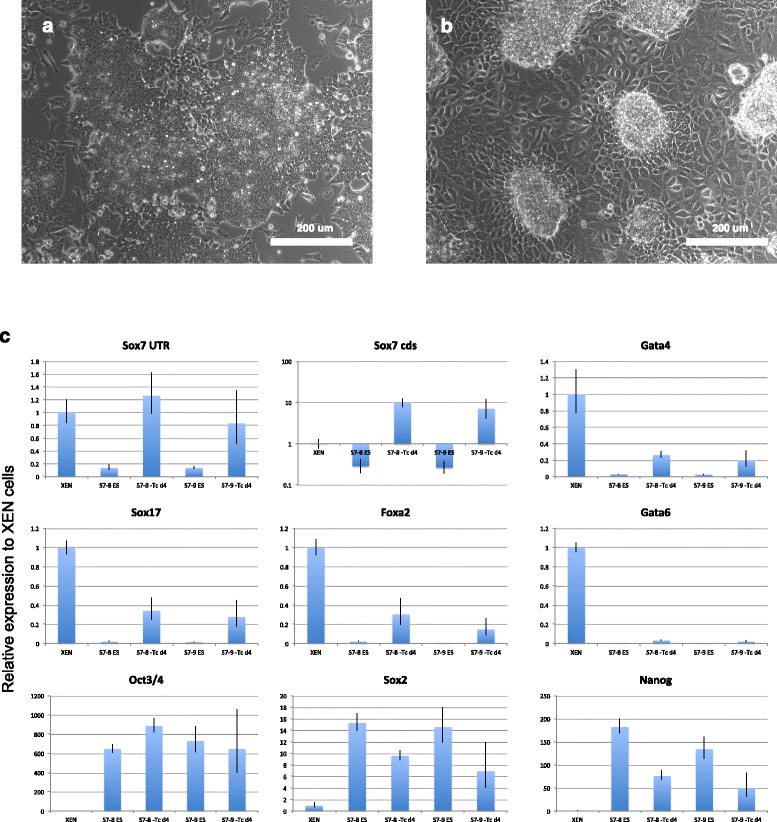
Effect of *Sox7* overexpression in ES cells. (**a**, **b**) ES cells carrying tetracycline-inducible *Sox7* transgene at the modified *Rosa26* locus are cultured for 4 days with (**a**) or without (**b**) tetracycline in the presence of LIF. Scale bar = 200 μm. (**c**) qPCR analysis of day 4 Sox7 expressing cells. Results are relative expression level to embryo-derived XEN cells and normalised to *Gapdh*. Two independent experiments by different clones (RTS7-8 and −9) are shown. Error bar represents standard deviation from triplicates

Since the assessment of the effect of overexpression of *Sox17* in mouse ES cells has been reported by several groupes [11, 16, 20–22], here we focused on the function of *Sox7*. We applied a tetracycline (Tc)-inducible gene expression system with Cre-mediated cassette-exchange [23], the Rosa-tet system, for the induction of *Sox7* transgene in ES cells. We previously confirmed that this system provides a moderate level of homogeneous transgene expression from the *Gt(ROSA)26Sor* locus upon withdrawal of Tc, which was sufficient for *Gata6* to induce differentiation to the primitive endoderm [23]. As a result, we found that *Sox7* over-expression using this system cannot make ES cells differentiate completely (Fig. 2a, b). Despite the total expression level of *Sox7* being about ten times higher than that of embryo derived XEN cells, these cells do not express comparable amount of primitive endoderm-associated TFs such as *Gata4, Gata6*, *Sox17* and *Foxa2*, and maintain the expression levels of pluripotency-associated genes (*Oct3/4, Sox2* and *Nanog*) at high levels (Fig. 2c). These data indicate that the impact of Sox7 overexpression in driving the primitive endoderm differentiation program is quite marginal, that is much weaker than Gata6.

### *Sox7* is not essential for the generation of primitive endoderm in ES cells

**Fig. 3:**
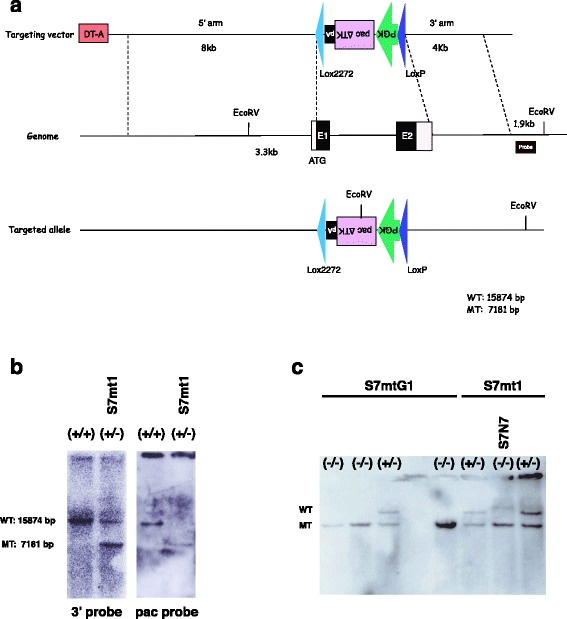
Generation of *Sox7*-null ES cells. (**a**) Design of the gene targeting vector. *Sox7* coding region including exon 1 and 2 were replace by PGK-pac∆TK casette flanked by *loxP* and *lox2722*. (**b**) Correctly targeted clone (S7mt1) was confirmed by Southern blot. (**c**) Genotyping results of high dose puromycin resistant clones by Southern blot. First five lanes are clones from S7mtG1 line (used in Fig. 5 and 6) and last three lanes are from S7mt1 line. We obtained three null lines from five S7mtG1 high dose puromycin resistance clones and two from six S7mt1 derivatives (half of the results is shown here)

Gain-of-function analysis of *Sox7* in ES cells suggested that it has a marginal impact on determining primitive endoderm fate compared to *Gata6*. To test whether it plays a physiological role in this process, we generated a *Sox7*-null ES cell line by gene targeting. The targeting vector was designed to remove the entire coding sequence spanning two exons. The first allele knockout clone was generated by the introduction of *Sox7* KO vector into EB3 ES cells followed by genotyping using Southern blot (Fig. 3a, b). Then one heterozygous clone, termed S7mt1, was selected with a high-dose of puromycin to obtain homozygous cells by a spontaneous gene conversion event [24] (Fig. 3c). As a result, we successfully established two *Sox7*-null ES cell lines (S7N4 and S7N7).

**Fig. 4:**
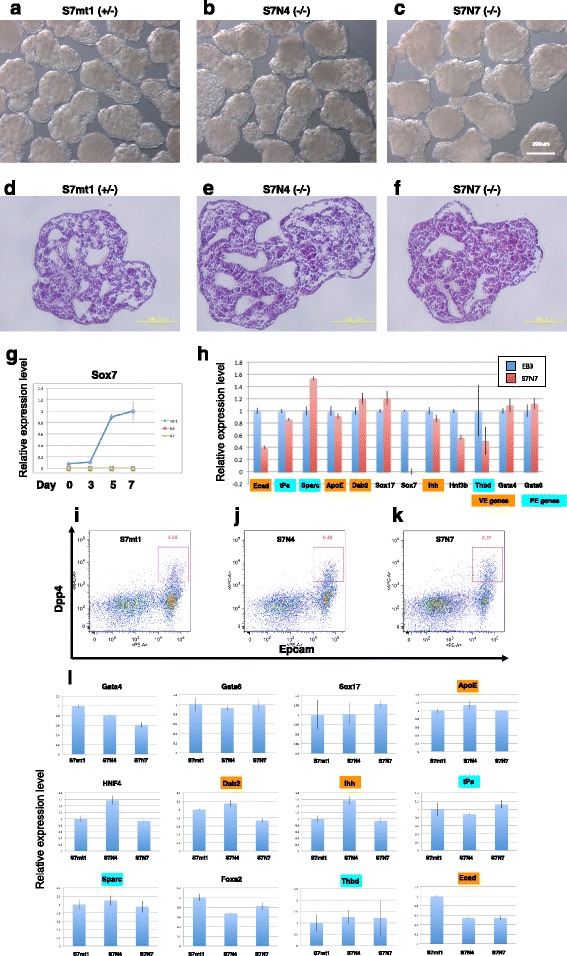
Differentiation of *Sox7*-null ES cells by EB formation. (**a**-**c**) Gross morphology of EBs at day 5 in each genotype indicated. Scale bar in C is 200 μm. (**d**-**f**) EBs in (a-c) were sectioned and stained with hematoxilyn and eosin. Scale bars are 100 μm. (**g**) Time course qPCR analysis of *Sox7* expression in Sox7-null clones (N4 and N7) and their parental heterozygote line (mt1) duirng EB formation. The relative expression levels normalized to *Gapdh* were shown with the error bar for standard deviation (n = 3). (**h**) qPCR analysis of gene expression in EBs at day 6. The relative expression levels normalized to *Gapdh* were shown with the error bar for standard deviation (n = 3). VE genes are marked in orange and PE genes are marked in blue. (**i**-**k**) VE fractions of EBs at day 7 were analyzed by FACS. Dead cells were eliminated by PI staining and living cells were separated by the staining with anti EpCam-PE and anti DppIV-APC. Double-positive fraction was indicated with its proportion (%). (**l**) qPCR analysis of VE fractions collected by FACS. Relative expression levels to S7mt1 parental line are presented. Expression levels were normalized to *Gapdh* with the error bar for standard deviation (n = 3)

These *Sox7*-null ES cells were morphologically normal and continued self-renewal in a manner similar to the parental EB3 ES cells. We then tested their ability to differentiate to extraembryonic endoderm by embryoid body (EB) formation assay. The EBs generated in a hanging drop culture of *Sox7*-null ES cells for 5 days were morphologically similar to those of parental ES cells and possessed the extra-embryonic endoderm layer at the surface (Fig. 4a-f). Next, we measured the expression of marker genes in these EBs by qPCR. During differentiation, *Sox7* was up-regulated in EBs derived from heterozygous S7mt1, but not in those derived from *Sox7*-null ES cells, S7N4 and S7N7 (Fig. 4g), confirming loss of *Sox7* in these mutant cell lines. When the expression levels of VE and PE marker genes were tested in these EBs, we found that all of them were properly expressed in EBs from *Sox7*-null ES cells at day 5, as they were in EBs from EB3 ES cells, indicating normal generation of PE and VE in the absence of *Sox7* (Fig. 4h). Next, we assessed the proportion of VE by FACS analysis for cell surface markers (Dpp4 and Epcam) that are expressed in VE but not in definitive endoderm [25]. We found that the proportion of the double positive fraction of Dpp4 and Epcam cells was similar in EBs of *Sox7-*null ES cells (Fig. 4i-k). We then collected this double positive fraction and analyzed the marker gene expression pattern. As shown in Fig. 4l, the expression level of the extraembryonic endoderm markers that we examined (which also include PE markers such as *Thbd, Sparc* and *tPa*) were not affected in both genotypes. These data show that the extraembryonic endoderm differentiation was almost completely unaffected by the deletion of *Sox7*.

**Fig. 5:**
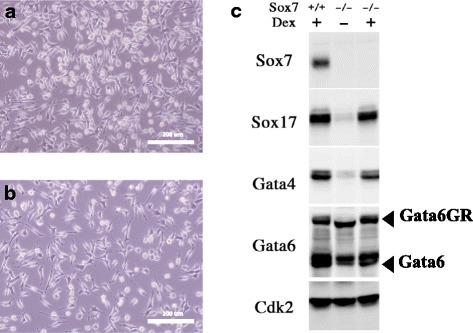
Differentiation of *Sox7*-null ES cells by Gata6GR. (**a**, **b**) The morphology of XEN-like cells induced by the activation of Gata6GR with Dex treatment for 4 days in *Sox7* (+/−) (**a**) and *Sox7* (−/−) (**b**) ES cells. Scale bars are 200 μm. (**c**) Western blot analysis of the expressions of the extraembryonic endoderm-associated transcription factors in XEN-like cells with or without *Sox7*

### *Sox7*-null ES cells can differentiate into XEN-like cells

As we showed above, *Sox7* is quickly up-regulated by the activation of G6GR in ES cells (Fig. 1). To test the function of *Sox7* in Gata6-induced differentiation toward XEN-like cells, we firstly introduced the *G6GR* expression cassette into S7mt1 (+/−) line (termed S7mtG1) and obtained *Sox7*-null ES cells from this parental line by high-dose puromycin selection (Fig. 3c). When these ES cells were cultured with Dex, they differentiated into XEN-like cells as efficiently as the parental S7mtG1 ES cells (Fig. 5a, b). Western blot analysis confirmed the proper induction of Sox17, Gata4 and Gata6 without *Sox7* in these XEN-like cells (Fig. 5c). Therefore, the morphological differentiation to primitive endoderm triggered by Gata6 does not require the function of *Sox7*.

### *Sox7* is not required for induction of primitive endoderm-specific gene expression profile by Gata6

**Fig. 6:**
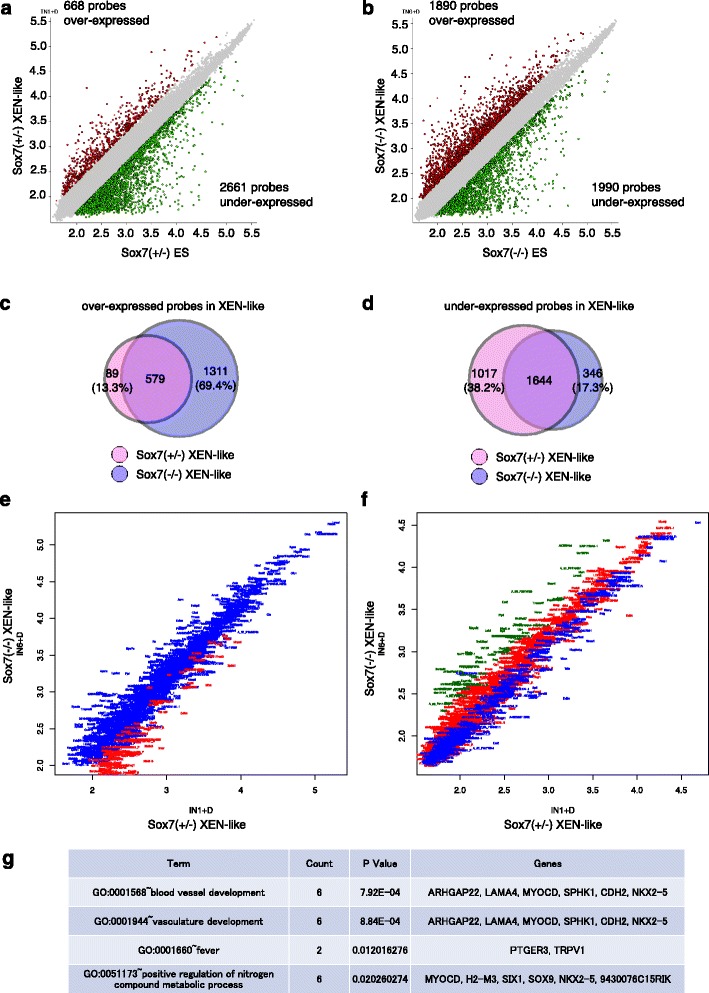
Microarray analysis of XEN-like cells with or without *Sox7.* (**a**, **b**) Scatter plots showing gene expression of ES cells (in X-axis) and XEN-like cells (in Y axis). The value is mean of log intensity from three replicates. Probes more than 2 fold change with FDR < 0.05 are shown. (**c**, **d**) Venn diagram showing the number of the probes which are over-lapped with each genotype. (**e**, **f**) Scatter plots created from the gene list (Additional file 2) identified in (**c**) and (**d**), respectively. Each probes was plotetted according to their gene symbol and *Sox7* (+/−) specific are shown in red and *Sox7* (−/−) are in blue. Greens in (**f**) show probes expresssed more than 3 fold. The value in both axis is mean of log intensity from three replicates. (**g**) The result of GO term analysis of biological process by using *Sox7* (+/−) 79 probes in (**c**). Top four from the lowest p-value GO terms are shown. IN1-D: *Sox7* (+/−) ES cells; IN1 + D: *Sox7* (+/−) XEN-like cells; IN6-D; *Sox7* (−/−) ES cells; IN6 + D; *Sox7* (−/−) XEN-like cells

To clarify the gene expression profile of *Sox7*-null XEN-like cells, we performed DNA microarray analysis. The results were analysed using the NIA array analysis tool [26]. Firstly, we identified differentially expressed genes (more than two fold, FDR < 0.05) before and after Dex treatment in *Sox7* (+/−) cells carrying Gata6GR (IN1) (Fig. 6a). Then, these genes were analysed in *Sox7* (−/−) cells carrying Gata6GR (IN6) to examine the differences (Fig. 6b, c and d). Comparison of 668 probes upregulated in *Sox7* (+/−) confirmed that 86.7 % of them (579 probes) were also up-regulated in *Sox7* (−/−) cells. This includes important TFs such as *Gata4, Foxa2* and *Sox17* as well as extraembryonic-endoderm genes such as *Fst, Sprac* and *Thbd* (Expression patterns of chosen extraembryonic endoderm genes are shown in Additional file 1: Figure S1). However, 69.4 % of the probes (1311 probes) which were highly expressed in *Sox7* (−/−) XEN-like compared to ES cells were not enriched in Sox7 (+/−) XEN-like cells (Fig. 6c) and only 61.7 % of down-regulated probes (1644 probes) in *Sox7* (+/−) overlapped with *Sox7* (−/−) (Fig. 6d). This suggests that loss of *Sox7* has little effect on the induction of extraembryonic endoderm genes, but its loss affects the magnitude of the down-regulation of ES-associated genes or hyper induction of extraembryonic endoderm-associated genes in this process.

Next, we re-plotted these differentially expressed genes to identify genes which are significantly up- or down-regulated in *Sox7* (−/−) XEN-like cells (Fig. 6e, f and Additional file 2). We confirmed that 79 probes out of 89 identified in Fig. 6c are under-expressed in *Sox7* (−/−) XEN-like cells (plotted in red in Fig. 6e), and that the 74 probes out of 1017 probes identified in Fig. 6d are more than 3 fold higher in *Sox7* (−/−) XEN-like cells than in *Sox7* (+/−) XEN-like cells (plotted in green in Fig. 6f). Among the *Sox7* (+/−) specific 89 probes, we could identify 22 genes which are not upregulated in *Sox7* (−/−) XEN-like cells (by visual inspections with the threshhold value in *Sox7* (−/−) XEN-like cells of 2.1, expresssion profile of these are shown in Additional file 3: Figure S2). These data suggest that Sox7 function is not required for the induction of most of the extra-embryonic endoderm-specific genes although it may involve in the down-regulation of the ES-associated genes. What is the nature of the 79 genes that fail to be up-regulated in Sox7 (−/−) XEN-like cells? When we performed GO term analysis for these 79 genes, we found that the significantly-enriched GO terms (P < 0.01) were blood vessel development, and vasculature development, confirming that the expression of the extraembryonic endoderm genes was not affected in the absence of *Sox7*.

## Discussion

Primitive endoderm is the second cell lineage segregated in mouse development and is essential for proper embryonic development. A recent report proposed a three step mechanism to direct differentiation of primitive endoderm [27]. The first step is the initiation of *Gata6* expression in the blastomeres of morula in a Fgf4-dependent manner at E2.5-E3.0. The second step is the competition between *Nanog* and *Gata6* to establish their reciprocal expression in epiblast and primitive endoderm, respectively, in the early blastocyst at E3.0-E3.5. The third step is the maturation of primitive endoderm by induction of *Gata4* and *Sox17* in a Fgf4-dependent manner in the late blastocyst at E3.5-E3.75. It was also reported that *Sox7* is up-regulated in primitive endoderm of the late blastocyst following the up-regulation of *Sox17* and *Gata4* [10], suggesting its role in finalizing the maturation step. Here we demonstrated that the function of *Sox7* is dispensable for both differentiation and maturation of primitive endoderm in an ES cell model system.

In human ES cells, it was shown that *Sox7* overexpression induces primitive endoderm differentiation [28]. However, here we showed that the inducible overexpression of *Sox7* is not sufficient to trigger rapid differentiation toward primitive endoderm, although the inducible expression of *Gata6* with the same system is sufficient [23]. These data are consistent with the scenario where Gata6 acts as a trigger of differentiation toward primitive endoderm whereas Sox7 acts later on. In the case of Sox17, several reports indicated that its forced expression in mouse ES cells causes their differentiation to primitive endoderm as well as XEN-like cells [11, 16, 20–22]. However, in these instances, the effect of Sox17 is limited to the induction of endoderm marker genes without a morphological differentiation event or gradual induction of differentiation to XEN-like cells after 12 days [22], and none of them demonstrated comparable activity of Sox17 to induce rapid differentiation to XEN-like cells within 4 days as in the cases for Gata4 and Gata6. Although we have not tested the ability of Sox7 to induce gradual differentiation, the previous data for Sox17 and the data presented in this manuscript for Sox7, suggest that the function of SoxF family member as a trigger of differentiation is distinct from that of Gata factors.

Recently, *Sox7* KO mice were generated to test its role in congenital diphragmatic hernia (CDH) development [13]. It was shown that, Sox7 null embryos died between E10.5 and E14.5, and that null embryos were indistinguishable from WT embryos at E8.5 stage. This result clearly shows that *in vivo Sox7* depletion does not affect the formation of extramebryonic endoderm tissues which supply developemental signals as well as nutrients to the embryo. It is consistent with our observations *in vitro* using *Sox7*-null ES cells, in which *Sox7* is dispensable for differentiation of extraembryonic endoderm by EB formation and by artificial activation of Gata6. In the analyses of gene expression induced by Gata6, we found that the extraembryonic endoderm-associated genes were properly up-regulated in the absence of *Sox7,* indicating that *Sox7* is not required for establishment of the extraembryonic endoderm-associated gene expression profile. Only 79 genes were activated in wild-type but not in *Sox7*-null XEN-like cells, but the GO term analysis revealed that these genes are enriched for vasculature development. This is an interesting coincidence, given the finding that *Sox7* is expressed in vascular endothelial cells in later development and *Sox7*-null embryos showed failure of yolk sac remodeling with signs of vascular failure [13]. This suggests an indispensable role of *Sox7* in vascular development both *in vivo* and *in vitro*.

Dispensability of the Sox7 function in extraembryonic endoderm development might be due to the functional overlap with similar genes. Sox7 belongs to the group F Sox family that includes Sox17 and Sox18 as the other members. It was reported that these three group F Sox family genes could have overlapping function. The loss of either Sox17 or Sox18 does not affect the extraembryonic endoderm development, as is the case for the loss of Sox7 [12, 29]. Interestingly, the analysis of knockout mice demonstrated the overlapping function of *Sox17* and *Sox18* in early cardiovascular development [30], and Hosking et al. presented that the effect of the loss of Sox18 in vascular development is compensated by Sox7 and Sox17 in particular genetic backgrounds, in which these genes are up-regulated in *Sox18*-null embryos although they are never expressed in wild-type embryos [29]. In primitive endoderm, Sox17 showed an overlapping expression pattern with Sox7, and *Niimi et al.* reported that both Sox7 and Sox17 activate the PE-specific enhancer of the *Lama1* gene, suggesting their overlapping function [31]. The comprehensive analysis of the group F Sox family members will be required to reveal their precise function in extraembryonic endoderm development.

## Conclusions

Sox7 was considered to be an important molecule for primitive endoderm differentiation because of its restricted expression pattern. However, this ES cell based study shows that *Sox7* (−/−) ES cells can differentiate into primitive endoderm lineage by spontaneous EB differentiation and Gata6-mediated XEN-like cell conversion. These results show that *Sox7* is dispensable for this lineage conversion event.

## Materials and methods

All experiments were performed according to the guidelines of RIKEN, Center for Developmental Biology with the approval of the RIKEN, Center for Developmental Biology institutional review board.

### Cell culture

All ES cell lines and XEN cell line used in this study were maintained in GMEM (Sigma-Aldrich) supplemented with 10 % FCS, 1x NEAA, 1 mM Sodium Pyruvate, 10^-4^M 2-mercaptoethanol, and 1,000 U/ml of LIF on gelatin coated dish. EB3 cell line was used to target *Sox7* locus. After the electroporation, selection was performed in the presence of 1.5 μg/ml of puromycine. High dose puromycine selection was performed at 22.5 μg/ml. *Gata6-GR* induced differentiation experiments were performed in the presence of 100 μM Dex with ES medium containing LIF. EB formation was performed in hanging drop culture at the concentration of 5,000 cells per drop without LIF.

### Plasmid vectors and introduction into ES cells

For Cre-recombinase mediate casette exchange, we cloned *Sox7* cDNA into *pZhc* vector (http://www.cdb.riken.jp/pcs/protocol/vector/vector_top.html). Lipofection was done by TransIT-LT1 (Mirus). In Rosa-tet system, 1.6 μg of *pZhc-Sox7* plasmid are cotransfected with 0.4 μg *pCAGGS-Cre* in 6-well plates and selceted clones in the presence of 20–40 μg/ml of Zeocin. Following drug selection, clones were chosen by the FACS analysis of Venus expression level when Tc was withdrawed. *Gata6-GR* is cloned into *pPBCAG-cHA-IN* vector and introduced 1.6 μg of them into ES cells by lipofection with 0.4 μg of *pCAG-PBase. Sox7* targeting vector was constructed by the method described before [32] with *pDONR-PGK-pac∆TK*. Targeting vector was designed for future casette exchange, so drug resistance casette was flanked with *loxP* and *lox2272* [33]. Oligo nucleotides sequence retrieving the targeting arm from BAC are A:5′- TTAGGGAAAGGAACATGGATCCTAAGTCTATGTCTCCAAATGGAGGGTCACAACTTTTCTATACAAAGTTGGCATTAT-3′, B: 5′-CAGGTCAGCGCCGGCCCCACGAGGCGAAGCCAAGTGACCCGCGTTCGGCCATAACTTCGTATAGGATACTTTATACGAAGTTATATGGCAAGTTTGTACA-3′, C: 5′-GAGACCTAGTATGAATTTAAAAAAAATACTATTTCAAAGGATAGAATGGTATAACTTCGTATAATGTATGCTATACGAAGTTATCCACTTTGTACAAGAA-3′ and D:5′-TCCATTTTCAAATGCCCTGGTCACCCCAGAGGCCCCAAGAAGGCAGTTATCAACTTTATTATACAAAGTTGGCATTAT-3′. Targeting vector was linearised by PmeI and electroporated at 800 V, 3 μF by Gene pulser (BioRad).

### Southern Blot

Genomic DNAs were digested with EcoRV and then transferred to nylon membrane. Sox7 3′ external probe was amplified with primers 5′-GAAAAGATAGGAATACCAG-3′ and 5′-AATGCAATCACAGTGAGACT-3′, and full length pac sequence was used as an internal probe.

### FACS analysis

Cells were analyzed and collected by FACS Aria (BD) with anti-DppIV antibody (R&D) and anti-EpCam antibody (eBioscience). Dead cells were eliminated by propidium iodide.

### Western blot

Western blot was done as described previously [34]. The antibodies used were goat anti-Gata4 (Santa Cruz), goat anti-Gata6 (R&D), goat anti-Sox7 (R&D), goat anti-Sox17 (R&D) and rabbit anti-Cdk2 (Santa Cruz).

### Quantitative RT-PCR

Primers and methods were followed as described previously [15]. Primers used for *Thbd* were 5′-CTTCTCCAAGTCCCTTCACG-3′ and 5′- CTGTGTTGCTAGCAGGTGGT-3′, and for *Sox7* cds were 5′-AGATGCTGGGAAAGTCATGG-3′ and 5′-GCTTGCCTTGTTTCTTCCTG-3′.

### Microarray analysis and GO term analysis

RNAs are isolated from ES cells and XEN-like cells at day 4. DNA microarray analyses were performed using a SurePrintG3 Mouse GE Microarray 8x60K (Agilent Technologies). Microarray results were analyzed uisng NIA Array Analysis Software. Complete array data will be available on the GEO (NCBI) website. GO term analysis was performed by DAVID (http://david.abcc.ncifcrf.gov) [35, 36].

## Availability of supporting data

The results of DNA microarray is available from NCBI gene expression omnibus, accesion number GSE66971.

## Additional files

Additional file 1: Figure S1. The expression pattern of extraembryonic endoderm genes with or without *Sox7* in Gata6 induced XEN-like cells. Additional file 2:
**Xlsx file containing the results of DNA microarray data.** Entire gene lists with expression values of *Sox7* (+/−) or (−/−) ES cell and XEN-like cells and gene lists with values used for generating Fig. 6e and f are included. *Sox7* (+/−) ES cells and XEN-like cells are designated as IN1-D and IN1 + D, *Sox7* (−/−) ES cells and XEN-like cells are designated as IN6-D and IN6 + D respectively. Additional file 3: Figure S2. Genes which failed to upregulated in *Sox7* (−/−) XEN-like cells.

## Abbreviations

ES: embryonic stem
PE: parietal endoderm
VE: visceral endoderm
TF: transcription factor
XEN: extraembryonic endoderm stem
GR: glucocorticoid receptor
Dex: dexamethasone
Tc: tetracyclin
EB: embryoid body
