# Supplementary figures and images for: Sox7 is dispensable for primitive endoderm differentiation from mouse ES cells
Source: BMC Dev Biol. 2015 Oct 16;15:37. doi: 10.1186/s12861-015-0079-4 (PMC4609079; doi:10.1186/s12861-015-0079-4)

Fig.S1 Kinoshita M et al

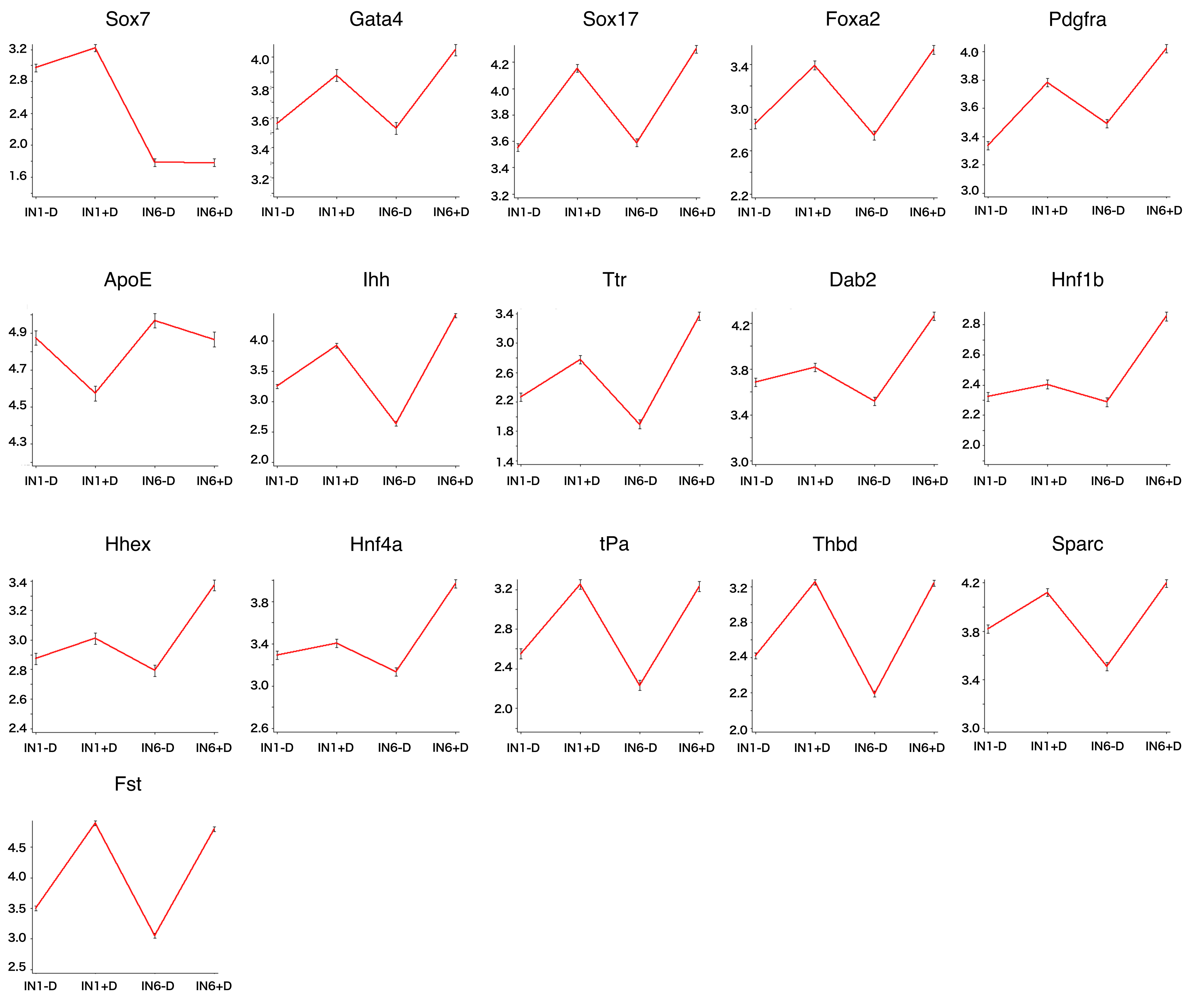

Supplement: Additional file 1: Figure S1. — The expression pattern of extraembryonic endoderm genes with or without Sox7 in Gata6 induced XEN-like cells. [file 12861_2015_79_MOESM1_ESM.pdf]

Fig.S2 Kinoshita M et al

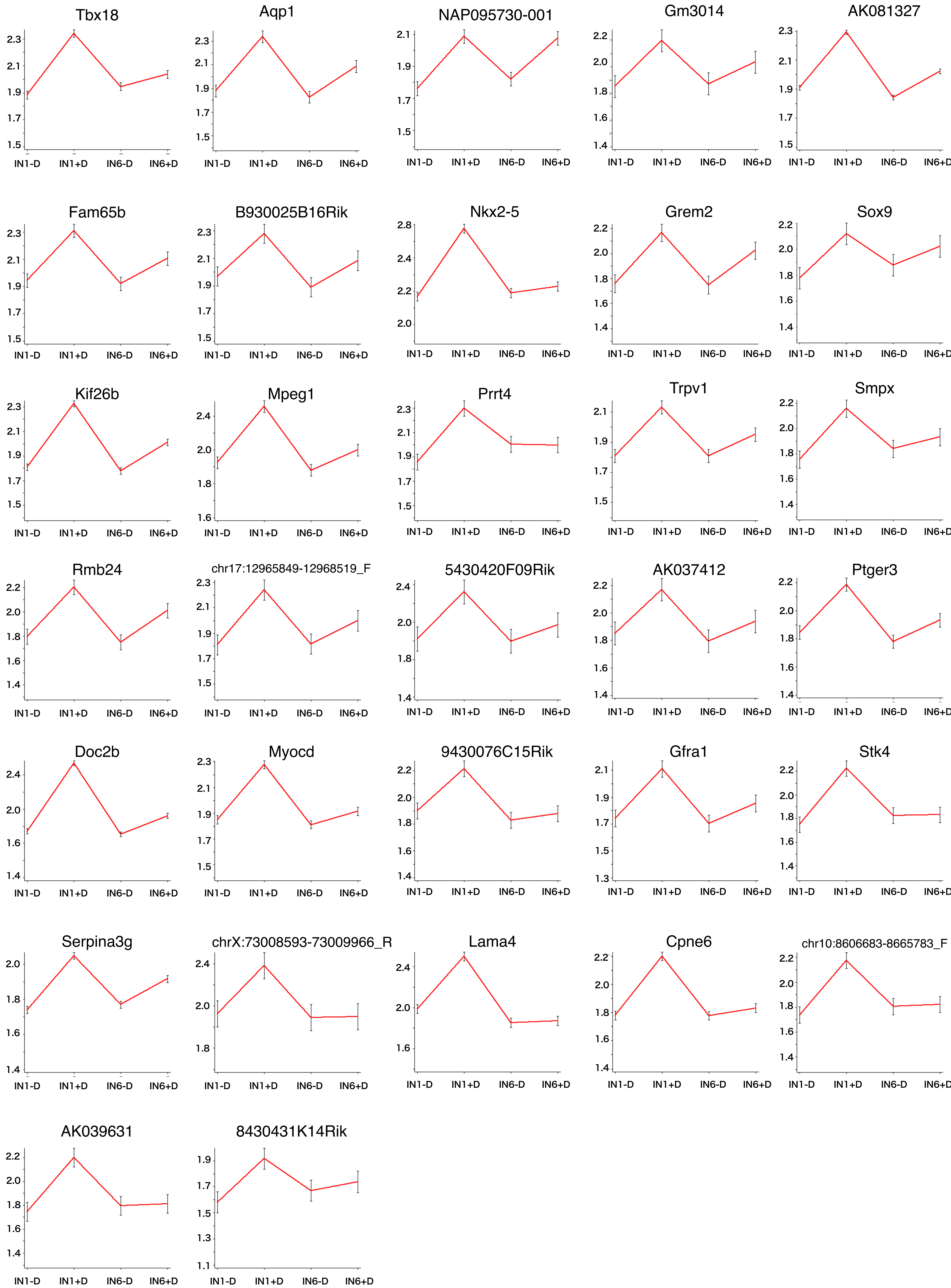

Supplement: Additional file 3: Figure S2. — Genes which failed to upregulated in Sox7 (−/−) XEN-like cells. [file 12861_2015_79_MOESM3_ESM.pdf]
